# Jarborandi Proposed as a Remedy in Hydrophobia

**Published:** 1878-05

**Authors:** 


Jaborandi Proposed as a Remedy in Hydrophobia.—J. G. Sinclair Coghill, M.D., F.R.C.P., Edinburgh (*British Medical Journal*, January 5th, 1878):

Dr. Coghill, from observations on cases of hydrophobia, concludes that, whereas, the salivary glands are the chief emunctories of the poison in the brute at least, and, whereas, the action of the salivary glands is suspended in man when attacked by this disease, therefore, to restore the functions to those glands, should be the first and especial aim of the medical attendant.

Reasoning thus, the doctor concludes that jaborandi, as being the most efficient sialogue and diaphoretic known, will most completely fill this indication. The drug should be given in full doses, with a view to producing its effects as speedily as possible. An infusion of a drachm of jaborandi to the ounce of water may be given per rectum, or a concentrated water extract may be given hypodermically.

